# Adherence to the Taiwan Daily Food Guide and the Risk of Type 2 Diabetes: A Populational Study in Taiwan

**DOI:** 10.3390/ijerph20032246

**Published:** 2023-01-27

**Authors:** Tsung-Ju Tsai, Ming-Chieh Li

**Affiliations:** 1 Department of Traditional Medicine, Wan Fang Hospital, Taipei Medical University, Taipei 11696, Taiwan; 2 Department of Health Promotion and Health Education, College of Education, National Taiwan Normal University, Taipei 10610, Taiwan; 3 Department of Public Health, College of Public Health, China Medical University, Taichung 406040, Taiwan

**Keywords:** Daily Food Guide, diet, vegetables, type 2 diabetes, HbA1c

## Abstract

This study aims to examine whether adherence to the Taiwan Daily Food Guide relates to the risk of type 2 diabetes. A population-based study was conducted using data from the Nutrition and Health Survey in Taiwan (NAHSIT) 2013–2016. Dietary intakes were assessed using a validated food-frequency questionnaire. Type 2 diabetes was defined as a fasting serum HbA1c level of 6.5% or higher or participants who have received treatment for type 2 diabetes or have reported a physician diagnosis of type 2 diabetes. A total of 2534 Taiwanese adults aged 19 and above were included. We found that the Daily Food Guide adherence was negatively associated with the prevalence of type 2 diabetes. The odds ratios (ORs) for those in the fourth quartile of the recommended total servings was 0.67 (95% confidence interval (CI) = 0.45–0.99) compared with those in the first quartile. In addition, those who were men (OR = 1.46, 95% CI = 1.07–1.98), aged more than 50 to 65 (OR = 6.48, 95% CI = 2.57–16.35), or more than 65 (OR = 6.81, 95% CI = 2.56–18.08), with body mass index (BMI) of 24 to less than 27 (OR = 2.08, 95% CI = 1.55–2.79), had BMI of more than 27 (OR = 3.63, 95% CI = 2.73–4.83), had an education level of junior high and high school (OR = 1.68, 95% CI = 1.03–2.74), were divorced, separated, widowed, or refused to answer (OR = 1.39, 95% CI = 1.03–1.88) were associated with an increased prevalence of type 2 diabetes. In conclusion, people who adhere better to the Taiwan Daily Food Guide were found to have a reduced risk of type 2 diabetes.

## 1. Introduction

Type 2 diabetes is a significant public health concern worldwide. Unfortunately, the global burden of diabetes mellitus continues to increase, particularly in developed countries and regions [1]. Additionally, approximately one-third of diabetes-related deaths occur in individuals under the age of 60, raising further concerns about the impact of the disease [2]. Type 2 diabetes not only reduces the quality of life and leads to morbidity and premature mortality [3] but also poses a significant economic burden, projected to increase from USD 1.3 trillion in 2015 to USD 2.1 trillion by 2030 [4]. Taiwan is no exception to this trend, with the age-standardized prevalence of diabetes increasing from 6.2% in 1993–1996 to 7.8% in 2005–2008, and reaching 8.8% in 2017 [5]. Given the global pandemic of diabetes and the severe consequences it causes, it is crucial to identify effective prevention strategies to combat the disease.

The incidence of type 2 diabetes has been on the rise in recent years, and several factors have been identified as contributing to this trend. Studies have suggested that unhealthy lifestyle choices, being overweight or obese, and the aging of the human population may all contribute to the increased burden of type 2 diabetes [6,7,8]. Furthermore, research indicates that dietary patterns also play a significant role in the development of type 2 diabetes [9]. For example, a systematic review and meta-analysis found that dietary patterns characterized by a high intake of vegetables, legumes, fruits, poultry, and fish were negatively associated with type 2 diabetes, while patterns characterized by high consumption of red and processed meats, refined grains, high-fat dairy, eggs, and fried products were positively associated with an increased risk of type 2 diabetes [10]. To sum up, the evidence suggests that a diet rich in plant-based foods may protect against developing type 2 diabetes.

Although adopting certain dietary patterns is associated with a decreased risk of type 2 diabetes, policymakers often prefer to promote general dietary guidelines rather than a specific dietary pattern. Similarly, in Taiwan, the most updated version of the Taiwan Daily Food Guide was released by the government in 2018, aimed to prevent nutrient deficiencies by ensuring that a diet provides at least 70% of the Dietary Reference Intakes (DRIs) and also considered evidence from epidemiological studies to reduce the risk of cardiovascular and metabolic diseases [11,12]. However, there is limited knowledge on how adherence to the Taiwan Daily Food Guide relates to disease status, including type 2 diabetes. Therefore, the aim of this study is to examine whether adherence to the Taiwan Daily Food Guide is associated with the risk of type 2 diabetes.

## 2. Materials and Methods

We conducted a population-based study using nationally representative data from the Nutrition and Health Survey in Taiwan (NAHSIT) 2013–2016. The objective of NAHSIT was to monitor the overall nutrition status of the Taiwanese people through detailed interviews and examinations. The details of the survey method can be found in the literature [11,13,14]. In brief, three-stage probability sampling covering 359 townships or city districts was conducted to select study participants. The Latin square design was used to consider seasonal variations in dietary consumption. Physical examinations, including measuring body mass index (BMI), were conducted at temporary health examination stations. Participants also underwent a face-to-face interview to collect demographic data such as age, sex, education, marital status, smoking and alcohol consumption habits, physical activity levels, and medical history.

The study participants were restricted to participants aged ≥ 19, corresponding to the definition of the Taiwan Daily Food Guide. This study was approved by the China Medical University & Hospital Research Ethics Center (CRREC-108-136). The NAHSIT participants gave informed consent for their data to be used for research purposes.

### 2.1. Defining Type 2 Diabetes Mellitus

Type 2 diabetes was defined as (1) a fasting serum HbA1c level of 6.5% or higher; (2) those who had received treatment for type 2 diabetes; or (3) those who have been diagnosed with type 2 diabetes.

### 2.2. Accessing the Taiwan Daily Food Guide Adherence

The Taiwan Daily Food Guide details were described elsewhere [11,15]. Briefly, in 2018, the Health Promotion Administration published the Taiwan Daily Food Guide, which contains six food groups: (1) cereals and whole grains; (2) protein-rich foods (soybean, fish, egg, and meat); (3) dairy products; (4) vegetables; (5) fruits; (6) fats, oils, and nuts. The guide recommends minimal servings of the six food groups based on one’s daily energy needs (Supplementary Table S1). Dietary intakes were accessed using a validated 79-item food-frequency questionnaire (FFQ) [16].

We constructed a Daily Food Guide index to measure the Daily Food Guide adherence of study participants. The details of the methods were described elsewhere [11,17,18]. Because the FFQ used in this study is not a validated method for assessing the intake of fat and oil, we decided not to assess the intake of fat and oil adherence. For each participant, the recommended quantity of each food group was estimated based on their estimated energy needs. Participants’ physical activity, healthy weight, and resting metabolic rate were utilized to calculate the estimated energy needs [11,12,19]. In the Taiwan Daily Food Guide, the level of physical activity is determined based on predefined criteria used to determine the recommended daily caloric intake for that person. For example, based on the statement, “Most of the time is spent sitting studying, doing homework, talking, and some of the time watching TV or listening to music, and there is about an hour spent on activities that require less energy consumption such as walking”, it can be inferred that the individual has low physical activity. In the Taiwan Daily Food Guide, participants are assigned a point based on their level of physical activity. For example, those with low physical activity would receive a score of 1.3, while those with high physical activity would receive a score of 1.9. This score is then used in the formula to calculate estimated energy needs: energy needs (kcal) = healthy weight (kg) × resting metabolic rate (kcal/kg/day) × level of physical activity. The adherence score for each food category was calculated according to the recommended number of servings for daily energy needs (Supplementary Table S1). For instance, if someone consumed exactly the recommended servings of a specific food group, they scored 1 for the specific food group; if someone consumed no serving of a specific food group, they received a score of 0. The score was calculated proportionately if someone consumed servings between zero and the recommended number or consumed servings higher than recommended.

### 2.3. Statistical Analysis

Descriptive analyses were conducted using Chi-square or Fisher’s exact test. We classified participants into categories defined by their adherence scores. Those who had a score higher than 6 were classified into group 5, meaning they had intakes greater than the recommended total servings from all food groups. The rest of the participants, with scores of or less than 1, were classified by the quartile of the adherence score.

We conducted multiple logistic regression models to investigate the association of adherence with the prevalence of type 2 diabetes. All logistic regression models were adjusted for age, sex, BMI, education level, alcohol intake, marital status, family income, smoking status, and physical activity. High physical activity was defined as engaging in high-intensity activity for at least 30 min per day, such as lifting heavy objects weighing 10 kg or more or jogging. Medium physical activity was defined as engaging in medium-intensity activity for at least 30 min per day, such as lifting objects weighing between 4.5 and 9 kg or brisk walking.

Study participants might change their dietary habits if they know they are diabetic. To reduce the temporal bias, we further conducted subgroup analyses by defining type 2 diabetes as an HbA1c level of 6.5% or more. In addition, we excluded those who had received treatment for type 2 diabetes or had been diagnosed with type 2 diabetes. All analyses were performed by SAS 9.4 (SAS Institute, Cary, NC, USA).

## 3. Results

A total of 2534 study participants were included in the final analyses without missing data. Of them, 386 had type 2 diabetes. Table 1 displays the demographic comparisons between those with or without type 2 diabetes. We found that those who have type 2 diabetes tended to be men (55.96% vs. 47.25%); to be older (age > 50: 90.16% vs. 56.00%); to have abnormal BMI (BMI ≥ 24: 72.28% vs. 44.79%); to be less educated (college or above: 8.55% vs. 23.93%); to be ever or current smokers (36.27% vs. 27.98%); to be either married or living with a partner (72.28% vs. 67.83%); to have lower family income (less than New Taiwan dollar (NT) $40,000: 38.86% vs. 27.66%); and to have low physical activity (57.51% vs. 35.57%).

The associations between the Taiwan Daily Food Guide adherence and the prevalence of type 2 diabetes are shown in Table 2. After controlling for potential confounders, the odds ratios (ORs) for those in the fourth quartile of the recommended total servings (group 4) was 0.67 (95% confidence interval (CI) = 0.45–0.99) compared with those in the first quartile (group 1). In addition, those who were men (OR = 1.46, 95% CI = 1.07–1.98), aged more than 50 to 65 (OR = 6.48, 95% CI = 2.57–16.35) or more than 65 (OR = 6.81, 95% CI = 2.56–18.08), with BMI of 24 to less than 27 (OR = 2.08, 95% CI = 1.55–2.79) or BMI more than 27 (OR = 3.63, 95% CI = 2.73–4.83), had an education level of junior high and high school (OR = 1.68, 95% CI = 1.03–2.74), and divorced, separated, widowed, or refused to answer (OR = 1.39, 95% CI = 1.03–1.88), were associated with an increased prevalence of type 2 diabetes.

Participants may change their dietary behavior if they know they are diabetic patients. To avoid the bias induced by the aforementioned issue, subgroup analyses were conducted by defining type 2 diabetes as an HbA1c level of 6.5% or higher and, at the same time, excluding those who had received treatment for type 2 diabetes or had been diagnosed with type 2 diabetes. The results are shown in Supplementary Table S2. Similar but more evident findings were observed. The OR for those in the fourth quartile of the recommended total servings (group 4) was 0.44 (95% CI = 0.25–0.80) compared with those in the first quartile (group 1).

In Table S3, we examined the relationship between individual food group adherence and the prevalence of type 2 diabetes. After controlling for potential confounders, we found that the adherence score for food group 5 (fruits) was negatively related to the risk of type 2 diabetes.

## 4. Discussion

In this study, we found that participants reporting better adherence to the Taiwan Daily Food Guide were associated with a reduced risk of having type 2 diabetes. Protective effects still existed when participants had intakes of more than the recommended servings, although it was not statistically significant.

Although dietary guidelines in the Daily Food Guide were designed based on nutritional health knowledge [20], rather limited epidemiologic studies have been conducted to investigate whether guideline adherence benefits health outcomes. It is also true in Taiwan. Although previous studies in Taiwan have identified the possible dietary patterns related to several health effects [13,16,21], little is known about how adherence to the Daily Food Guide relates to diseases. The recommendations, in total, may be beneficial for certain diseases but not for others [22]. In Taiwan, the prevalence of diabetes was around 9.8% and ranked second in health insurance spending [23]. Since dietary intake plays an important role in the pathogenesis of type 2 diabetes [24], it is important to determine whether the national Daily Food Guide is potentially beneficial for diabetic management. Our findings provide preliminary evidence that adherence to the Taiwanese Daily Food Guide might provide benefits for type 2 diabetes. In addition, although we did not aim to examine the effects of individual food groups on the risk of type 2 diabetes, we found a negative relation between intakes of fruits and the risk of type 2 diabetes, consistent with previous findings [10]. Participants consuming vegetables higher than recommended servings also had a lower risk of type 2 diabetes, but the finding was not statistically significant.

The association between dietary patterns and the development of type 2 diabetes has been extensively investigated in the literature. For example, the Mediterranean diet, characterized by high consumption of fruits, vegetables, legumes, and fish, is associated with a reduced risk of type 2 diabetes [24,25,26]. Furthermore, a systematic review and meta-analysis found that dietary patterns high in vegetables, legumes, fruits, poultry, and fish were negatively associated with type 2 diabetes, whereas patterns high in red and processed meats, refined grains, high-fat dairy, eggs, and fried products were positively associated with an increased risk of type 2 diabetes [10]. Studies in Asia have also provided evidence for beneficial dietary patterns concerning the prevention of type 2 diabetes. For instance, a study in Japan found that a dietary pattern characterized by higher consumption of vegetables, potatoes, seaweeds, fruits, and soybean products was related to a lower incidence of type 2 diabetes [27]. Additionally, a study conducted in Singapore found that a higher intake of vegetables, fruits, and soy foods was associated with a decreased risk of type 2 diabetes [28]. Another study in China found that consuming potatoes, beef and mutton, fresh fruits, freshly squeezed juice, and soy products was associated with the onset of type 2 diabetes [29]. In conclusion, the literature suggests that dietary patterns characterized by high intake of plant-based foods, such as fruits, have protective effects against developing Type 2 Diabetes, consistent with our findings.

In the current study, we also found that men, older participants, those with higher BMI, and those with lower education levels had an increased risk of type 2 diabetes, consistent with previous studies [30,31,32,33]. Those who were divorced, separated, widowed, or refused to answer also had an increased risk of type 2 diabetes. The underlying mechanism for the difference in effects on dietary habits between “single” and “divorced, separated, or widowed” is poorly understood. However, previous studies have shown that marital transitions can influence dietary and other health behaviors [34]. Single individuals do not experience marital transitions and may not change their dietary or health behaviors as a result. We also found no association between physical activity, family income, smoking status, and alcohol consumption with type 2 diabetes, which contradicts previous studies. This discrepancy may be due to the cross-sectional design of the study, which could lead to the misclassification of covariates and bias toward the null hypothesis. 

The present study has some strengths and weaknesses. Strengths include a sufficient sample size to examine the effects of the Taiwan Daily Food Guide on type 2 diabetes and a population-representative sample that allows for the generalization of the findings to a wider population. Weaknesses include: (1) The cross-sectional design of the study precludes determination of the causal direction of the association between adherence level and type 2 diabetes. However, in subgroup analyses, we excluded participants who had already been diagnosed with or treated for type 2 diabetes and found similar findings. This suggests that the reverse relationship between type 2 diabetes influencing dietary intake is unlikely to occur. (2) The current study only assessed dietary intake at one time point, which may introduce non-differential misclassification of exposures and outcomes. However, non-differential misclassification of exposure typically biases towards the null, which would result in an underestimated effect. (3) The Taiwan Daily Food Guide also includes recommendations for fat and oil intake, but our study was unable to assess their impact on type 2 diabetes due to limitations in the dietary assessment tool used. Previous studies have shown an inverse association of ruminant trans-palmitoleic acid [35] and vegetable fat [36] with the risk of type 2 diabetes. This limitation in our study should be considered when interpreting the findings.

In conclusion, the current study found that participants reporting better adherence to the Taiwan Daily Food Guide in total were associated with a reduced risk of having type 2 diabetes. Additionally, the study found that men, older participants, those with higher BMI, and those with lower education levels had an increased risk of type 2 diabetes. Furthermore, we also found that a higher intake of fruits may have protective effects against developing type 2 diabetes. More prospective cohort studies are warranted to confirm our findings.

## Figures and Tables

**Table 1:** Demographic descriptions of included participants. (*n* = 2534).

	Participants without Type 2 Diabetes	Participants with Type 2 Diabetes	
Variables	*n* = 2148	*n* = 386	*p*-Value
Sex			
Men	1015 (47.25%)	216 (55.96%)	<0.01
Women	1133 (52.75%)	170 (44.04%)	
Age			
Age ≤ 30	346 (16.11%)	7 (1.81%)	<0.0001
40 ≥ age > 30	273 (12.71%)	6 (1.55%)	
50 ≥ age > 40	326 (15.18%)	25 (6.48%)	
65 ≥ age > 50	650 (30.26%)	160 (41.45%)	
Age > 65	553 (25.74%)	188 (48.71%)	
Body mass index			
BMI < 24	1186 (55.21%)	107 (27.72%)	<0.0001
27 > BMI ≥ 24	522 (24.30%)	120 (31.09%)	
BMI ≥ 27	440 (20.49%)	159 (41.19%)	
Education level			
Elementary school or lower	1106 (51.49%)	165 (42.75%)	<0.0001
Junior high and high school	528 (24.58%)	188 (48.70%)	
College or above	514 (23.93%)	33 (8.55%)	
Current alcohol intake			
No	996 (46.37%)	191 (49.48%)	0.0746
Rarely	881 (41.01%)	136 (35.23%)	
Frequently	271 (12.62%)	59 (15.28%)	
Smoking status			
Never smoked	1547 (72.02%)	246 (63.73%)	<0.001
Former smoker	286 (13.31%)	81 (20.98%)	
Current smoker	315 (14.67%)	59 (15.29%)	
Marital status			
Single	407 (18.95%)	14 (3.63%)	<0.0001
Married or lived together	1457 (67.83%)	279 (72.28%)	
Divorced, separated, widowed, or refused to answer	284 (13.22%)	93 (24.09%)	
Family income			
<NT $10,000	161 (7.50%)	49 (12.69%)	<0.0001
NT $10,000 ≤ Income < NT $40,000	433 (20.16%)	101 (26.17%)	
NT $40,000 ≤ Income < NT $80,000	550 (25.61%)	78 (20.21%)	
>NT $80,000	524 (24.39%)	60 (15.54%)	
Don’t know or refuse to answer	480 (22.34%)	98 (25.39%)	
Physical activity			
Low	764 (35.57%)	222 (57.51%)	<0.0001
Median	1325 (61.69%)	157 (40.67%)	
High	59 (2.74%)	7 (1.82%)	

NT: New Taiwan dollars. In 2016, the minimum wage in Taiwan was TWD 126 per hour.

**Table 2:** Associations of the Taiwan Daily Food Guide adherence and the prevalence of type 2 diabetes.

Variables	*n* = 2534
Diet score group	
Total scores equal to or lower than recommendation	
Group 1	Reference
Group 2	0.75 (0.51–1.11)
Group 3	0.55 * (0.36–0.84)
Group 4	0.67 * (0.45–0.99)
Total scores higher than recommendation	
Group 5	0.81 (0.56–1.17)
Sex	
Women	Reference
Men	1.46 * (1.07–1.98)
Age	
Age ≤ 30	Reference
40 ≥ age > 30	0.80 (0.25–2.58)
50 ≥ age > 40	2.43 (0.91–6.46)
65 ≥ age > 50	6.48 * (2.57–16.35)
Age > 65	6.81 * (2.56–18.08)
Body mass index	
BMI < 24	Reference
27 > BMI ≥ 24	2.08 * (1.55–2.79)
BMI ≥ 27	3.63 * (2.73–4.83)
Education level	
College or above	Reference
Junior high and high school	1.68 * (1.03–2.74)
Elementary school or lower	1.25 (0.81–1.93)
Current alcohol intake	
No	Reference
Rarely	0.95 (0.72–1.24)
Frequently	0.90 (0.62–1.31)
Smoking status	
Never smoked	Reference
Former smoker	1.10 (0.77–1.57)
Current smoker	1.04 (0.71–1.52)
Physical activity	
Low	Reference
Median	0.82 (0.59–1.15)
High	1.18 (0.47–2.94)
Marital status	
Married or lived together	Reference
Single	0.70 (0.35–1.40)
Divorced, separated, widowed, or refused to answer	1.39 * (1.03–1.88)
Family income	
<NT $10,000	Reference
NT $10,000 ≤ Income < NT $40,000	1.07 (0.71–1.63)
NT $40,000 ≤ Income < NT $80,000	1.08 (0.69–1.69)
>NT $80,000	1.08 (0.66–1.77)
Don’t know or refuse to answer	1.08 (0.71–1.64)

*: *p*-Value < 0.05; NT: The New Taiwan dollars; BMI: Body mass index; All logistic models were adjusted for age, sex, BMI, education level, alcohol drinking, smoking status, physical activity, marital status, and family income. Those with a score higher than 6 were classified into group 5, meaning they had intakes greater than the recommended servings in total from all food groups. The rest of the participants with scores of or less than 1 were classified by the quartile of the adherence score.

## Data Availability

Data from the Nutrition and Health Survey in Taiwan (NAHSIT) are available for research purposes, but an application must be submitted to and approved by the Ministry of Health and Welfare in Taiwan.

## Supplementary Materials

The following supporting information can be downloaded at: https://www.mdpi.com/article/10.3390/ijerph20032246/s1, Table S1: The Daily Food Guide for adults in Taiwan; Table S2: Associations between the Daily Food Guide adherence score and the prevalence of type 2 diabetes; Table S3: Associations between the levels of adherence to individual food group index and the prevalence of type 2 diabetes.
