# Supplementary material for: Adherence to the Taiwan Daily Food Guide and the Risk of Type 2 Diabetes: A Populational Study in Taiwan
Source: Int J Environ Res Public Health. 2023 Jan 27;20(3):2246. doi: 10.3390/ijerph20032246 (PMC9915098; doi:10.3390/ijerph20032246)
Supplement: Supplementary file 1 [file ijerph-20-02246-s001.zip › ijerph-2121678-supplementary.pdf]

Table S1. The Daily Food Guide for adults in Taiwan.

Index item	Daily energy needs (kcal/day)						
	≥ 1200	≥ 1500	> 1800	≥ 2000	≥ 2200	≥ 2500	> 2700
Minimal servings from cereals and whole grains	1.5	2.5	3	3	3.5	4	4
Minimal servings from protein-rich foods	3	4	5	6	6	7	8
Minimal servings from dairy products	1.5	1.5	1.5	1.5	1.5	1.5	2
Minimal servings from vegetables	3	3	3	4	4	5	5
Minimal servings from fruits	2	2	2	3	3.5	4	4
Minimal servings from fats, oils, and nuts							
Fats and oils	3	3	4	5	5	6	7
Nuts	1	1	1	1	1	1	1

Table S2. Associations between the Daily Food Guide adherence score and the prevalence of type 2 diabetes.

**Variables**	***n* = 2,242**
**Diet score group**	
**Total scores equal to or lower than the recommendation**	
Group 1	**Reference**
Group 2	0.70 (0.41-1.21)
Group 3	0.36 (0.19-0.68)
Group 4	0.44 (0.25-0.80)
**Total scores higher than the recommendation**	
Group 5	0.60 (0.36-1.02)
**Sex**	
Women	**Reference**
Men	1.52 (0.96-2.41)
**Age**	
Age ≤ 40	**Reference**
50 ≥ age > 40	2.50 (1.00-6.26)
65 ≥ age > 50	4.46 (1.92-10.34)
Age > 65	4.79 (1.82-12.56)
**Body mass index**	
BMI < 24	**Reference**
27 > BMI ≥ 24	3.24 (2.02-5.19)
BMI ≥ 27	5.40 (3.41-8.57)
**Education level**	
College or above	**Reference**

Junior high and high school	1.24 (0.61-2.54)
Elementary school	1.19 (0.65-2.19)
**Current alcohol intake**	
No	**Reference**
Rarely	1.00 (0.66-1.49)
Frequently	0.80 (0.45-1.42)
**Smoking status**	
No	**Reference**
Ever smoke	1.18 (0.70-2.01)
Current smoke	1.12 (0.65-1.93)
**Physical activity**	
Low	**Reference**
Median	1.00 (0.60-1.65)
**Marital status**	
Married or lived together	**Reference**
Single	0.74 (0.31-1.76)
Divorced, separated, widowed, or refused to answer	1.45 (0.91-2.29)
**Family income**	
Income < NT $10,000	**Reference**
NT $10,000 ≤ Income < NT $40,000	0.77 (0.40-1.46)
NT $40,000 ≤ Income < NT $80,000	0.87 (0.44-1.70)
Income > NT $80,000	0.98 (0.48-2.00)
Don't know or refuse to answer	1.01 (0.54-1.88)

NT: The New Taiwan dollars; BMI: Body mass index; All logistic models were adjusted for age, sex, BMI, education level, alcohol drinking, smoking status, physical activity, marital status, and family income. Type 2 diabetes was defined as a serum HbA1c level of 6.5% or higher and at the same time excluded participants who have received treatment for type 2 diabetes or with a self-reported physician diagnosis of type 2 diabetes.

Table S3. Associations between the levels of adherence to individual food group index and the prevalence of type 2 diabetes.

**Variables**	***n* = 2,242**
Food group 1 (whole grains and cereals)	
Score<0.5	Reference
0.5<=Score<1	1.29 (0.68-2.44)
Score>1	2.12 (0.73-6.14)
Food group 2 (protein-rich foods)	
Score<0.5	Reference
0.5<=Score<1	1.41 (0.96-2.08)
Score>=1	1.23 (0.59-2.56)
Food group 3 (dairy products)	
Score<0.5	Reference
0.5<=Score<1	1.11 (0.72-1.72)
Score>=1	0.41 (0.05-3.15)
Food group 4 (vegetables)	
Score<0.5	Reference
0.5<=Score<1	0.78 (0.46-1.30)
Score>=1	0.83 (0.47-1.48)
Food group 5 (fruits)	
Score<0.5	Reference
0.5<=Score<1	0.55 (0.28-1.06)
Score>=1	0.43 (0.25-0.74)
Food group 6 (nuts)	
Score<0.5	Reference
0.5<=Score<1	0.64 (0.38-1.08)
Score>=1	0.53 (0.07-4.13)

Score = 1 means that the participants consumed exactly recommended servings of the food group; Score = 0.5 means that the participants consumed half of recommended servings of the food group.
